# Tunable directional subwavelength acoustic antenna based on Mie resonance

**DOI:** 10.1038/s41598-018-27970-5

**Published:** 2018-07-03

**Authors:** Jin Zhang, Ying Cheng, Xiaojun Liu

**Affiliations:** 1 Key Laboratory of Modern Acoustics, Department of Physics and Collaborative Innovation Center of Advanced Microstructures, Nanjing University, Nanjing, 210093 China; 2 State Key Laboratory of Acoustics, Institute of Acoustics, Chinese Academy of Sciences, Beijing, 100190 China

## Abstract

Modulating the emission pattern of classic sound sources with a sub-wavelength scale dimension is a challenging. In this letter, we demonstrate theoretically and experimentally that a Mie-resonance based antenna can be designed in various modes to effectively enhance the emissivity of the radiated power and directivity of radiation pattern. A 2.33-fold enhancement of the radiated power and an 8.93-fold enhancement of the sound intensity are achieved in the mainlobe direction. Furthermore, we propose reconfigurable antenna scanning where the selectable beam direction is free to be controlled from 0° to 360°. The tunable directional acoustic antenna offers a new way to control sound with the improved performance.

## Introduction

Recently, there has been considerable efforts to control the emission pattern of sound waves for its potential applications in various areas, such as focused sound waves for medical imaging, non-destructive testing of materials, etc^1–6^. However, most current acoustic sources with dimensions much smaller than the working wavelength exhibit fundamentally non-directional radiation pattern and emit wide-angled sound energy in almost all directions^3^. Traditional approaches to improve directivity mostly relied on modifying acoustic sources, such as phase arrays^4,5^ and Bessel beam patterns^6^. In these cases, the dimension of the sources is comparable to the wavelength and hence it still poses a fundamental barrier on radiation of directional sound waves by a single finite-size source. Therefore, using micro-devices in sub-wavelength scales to control acoustic radiation pattern is still a challenging but promising problem.

On the other hand, artificial structured metamaterials offer great opportunity to the manipulation of sound fields^7–31^. Phononic crystals have already been developed for controlling wave propagation based on the features of anisotropy^7,8^, antiresonance^9,10^, diffraction^11,12^, and band-edge state^13–15^. Recent success in the fabrication of sub-wavelength high-refractive-index particles also provides ideal candidates for multifunctional elements in acoustic devices. Apart from acoustic focusing^18,19^, it can be used to construct acoustic cloaking^20–22^, which possesses low-reflection and power-flow bending properties for different shapes of wavefront. Other promising applications include the imaging with a sub-wavelength resolution^23,24^ and the extraordinary acoustic transmission^25^. Moreover, this methodology can also be applied to obtain acoustic directivity and enhance emission using the acoustic Purcell effect then it becomes a powerful approach for antenna design^26,27^. In this letter, we achieve the unidirectional and bidirectional radiation patterns of low-frequency sound waves with a high emissivity by coupling a single point-like sound source with a micro Mie resonance-based antenna. For this configuration, a 2.33-fold enhancement of the radiated power and an 8.93-fold enhancement of the sound intensity are demonstrated in desired mainlobe direction. Compared with the other methods of acoustic antenna, the Mie-resonance-based antenna has some unique superiorities, including high emissivity, directional radiation, and reconfiguration flexibility.

## Results

### Design of the Mie-resonance based antenna

**Figure 1:**
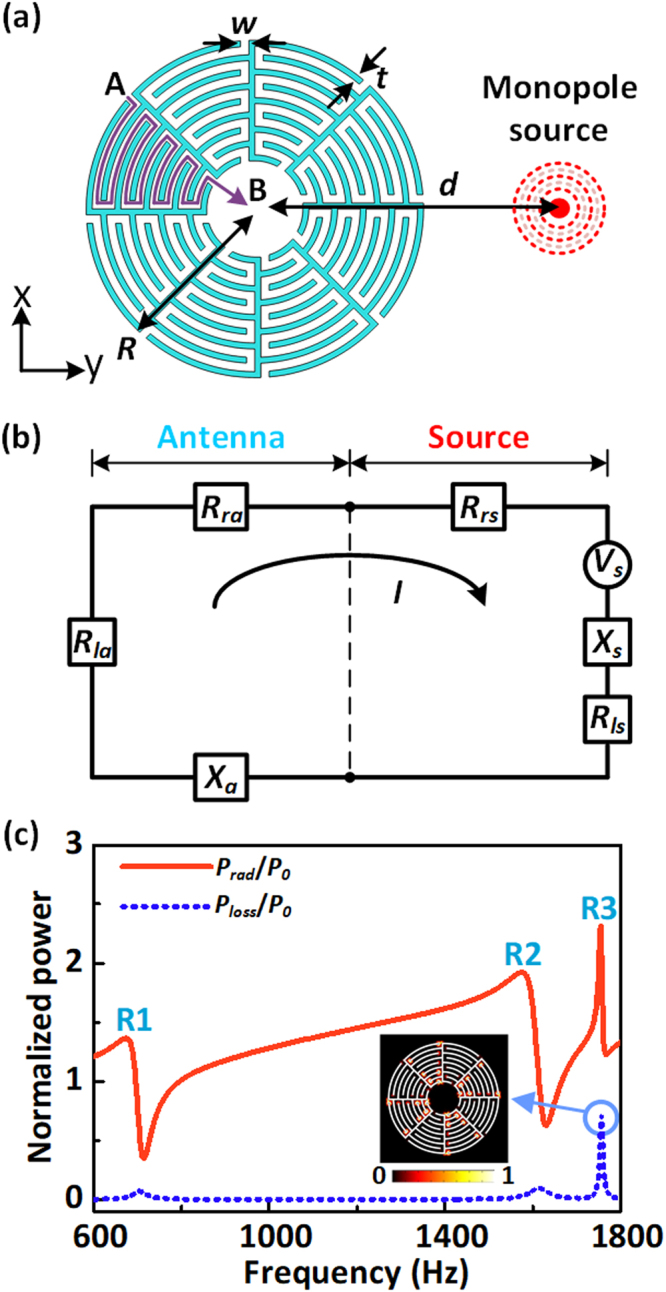
Concept to design the acoustic Mie-resonance based antenna (**a**) Schematics of the Mie-resonator-based antenna. An acoustic point source, which emits sound waves equally along all directions, is placed adjacent to a sub-wavelength maze-like Mie resonant unit. (**b**) Corresponding equivalent circuit. (**c**) Normalized radiated power and dissipated power for the antenna.

As schematically shown in Fig. 1(a), a classic acoustic monopole source, which emits sound waves equally in all directions, is placed close to a sub-wavelength Mie resonant unit with a maze-like structure. The distance between the center of the Mie resonator and the monopole source is *d* = 40 mm. The maze-like structure with a radius *R* = 30 mm and a wall thickness *t* = 0.035*R* is uniformly divided into eight sections, with each section having a zigzag channel with an identical width *w* = 0.05*R* and a curling number *N* = 8. Here *N* is defined as how many times the acoustic waves circulate in a zigzag channel. In a microscopic view, the labyrinthine structure forces the acoustic waves to travel in the folded channels rather than along the straight line from point A to B. Consequently, the path length of the waves is multiplied so that it can possess a high relative refractive index *n*_*r*_ dominated by the curled factor, which is the ratio between the total path length and the size of the unit cell.

### Equivalent circuit theory

We start from the intensive interactions between the point monopole source and the Mie resonator with monopolar, dipolar and multipolar modes, which leads to the enhanced excitation efficiency of the proposed antenna. The acoustic equivalent circuit in Fig. 1(b) illustrates the underlying physical mechanism of the antenna. *R*_*rs*_, *R*_*ls*_ and *X*_*s*_ represent the radiation resistance, loss resistance and the reactance of the sound source, respectively. The radiation resistance *R*_*rs*_ characterizes the radiation ability of the sound source into free space. Thus, the power emitted by the sound source alone in the absence of the Mie resonator antenna is given by^32^

1 $${P}_{0}=\frac{1}{2}{|I|}^{2}{R}_{rs}=\frac{{|{V}_{s}|}^{2}}{2}[\frac{{R}_{rs}}{{({R}_{rs}+{R}_{ls})}^{2}+{{X}_{s}}^{2}}],$$

where *V*_*s*_ is the equivalent voltage of the source and *I* is the current developed in the loop. For the antenna system, the Mie resonator should introduce the additional input impedance containing radiation resistance *R*_*ra*_, loss resistance *R*_*la*_, and reactance *X*_*a*_. Partial power emitted by the source is re-radiated through the mechanism provided by the radiation resistance, and the remaining power is dissipated which influences the overall efficiency of the antenna. The power delivered to the antenna for radiation is given by

2 $${P}_{rad}=\frac{1}{2}{|I|}^{2}({R}_{ra}+{R}_{rs})=\frac{{|{V}_{s}|}^{2}}{2}[\frac{{R}_{ra}+{R}_{rs}}{{({R}_{ra}+{R}_{la}+{R}_{rs}+{R}_{ls})}^{2}+{({X}_{a}+{X}_{s})}^{2}}],$$

and that for dissipation can be expressed as

3 $${P}_{loss}=\frac{1}{2}{|I|}^{2}{R}_{la}=\frac{{|{V}_{s}|}^{2}}{2}[\frac{{R}_{la}}{{({R}_{ra}+{R}_{la}+{R}_{rs}+{R}_{ls})}^{2}+{({X}_{a}+{X}_{s})}^{2}}]\cdot $$

Thus, the acoustic antenna can modulate the emissivity of the sound source and the power radiated into free space by adding an input impedance to the circuit. The input impedance of the antenna system is generally a function of frequency and depends on many factors including its geometry, method of excitation, and proximity to surrounding objects.

### Radiation performance of the proposed antenna

Figure 1(c) shows the spectra of the normalized radiated power *P*_rad_/*P*_0_ and dissipated power *P*_loss_/*P*_0_ for the acoustic antenna. The radiated power spectrum exhibits three peaks R1, R2 and R3 at *f* = 673 Hz, 1575 Hz and 1755Hz, respectively, suggesting that the emission is resonantly enhanced around these frequency regions. The dissipated power spectrum also exhibits three peaks at roughly the same frequencies as R1, R2 and R3. However, the dissipated power is always much less than the radiated power so that the antenna shows enhanced high radiation efficiency. It should be noted that the normalized radiated power and dissipated power both reach their maximum values of 2.33 and 0.72 at resonance R3, indicating that the enhancement of emissivity of the sound source is the strongest. The simulation is performed by the thermo-viscous module in COMSOL to characterize the acoustic response of the Mie resonator in a general viscous and thermally conduction fluid. The energy loss is mainly caused by the viscous power dissipation and the field distribution of viscous power dissipation for R3 is shown in the inset of Fig. 1(c). The loss appears mainly in the corner of the channel, indicating that the folded channel should lead to the viscous power loss at the resonance frequency although the previous research has demonstrated that a higher curling number allows structures with the same radius to exhibit much lower resonant frequency^2,28^.

**Figure 2:**
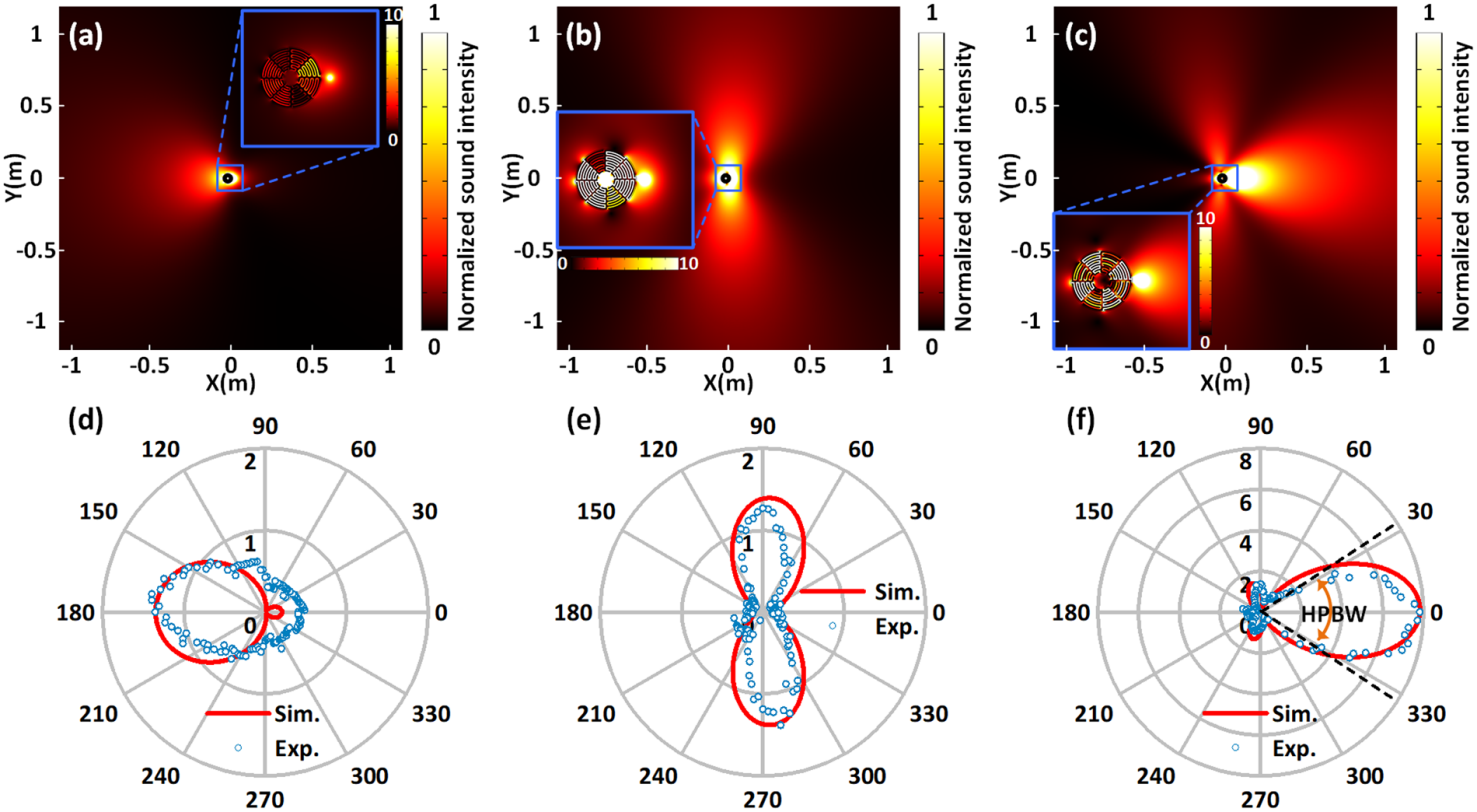
Radiation performance of the antenna. Simulated field distributions of sound intensity in the *xy*-plane for the antenna at resonances for (**a**) R1, (**b**) R2, and (**c**) R3. The corresponding 2D polar plots of simulated and measured far-field radiation pattern are shown in (**d**–**f**).

We further confirm the directional emission behavior of the multimode system. The distributions of sound intensity field induced by the antenna at resonances R1, R2 and R3 are shown in Fig. 2(a–c), respectively. The light area is the beam or main lobe, while the darker lines fanning out around it are side lobes. When the Mie resonator works in monopole mode, dipole mode and quadrupole mode at resonance R1, R2 and R3 [see insets of Fig. 2(a–c)], the antenna exhibits backward unidirectional radiation, lateral bidirectional radiation, and forward unidirectional radiation, respectively. These results clearly confirm that the radiation peaks of Fig. 1(c) are associated with the excitation of Mie resonances on the antenna. The maximum sound intensity inside the antenna at resonance R3 is much higher than that at resonance R2 and R1, indicating that the quadrupole mode has the strongest ability to localize the acoustic energy. Consequently, the radiated power and the loss power of R3 resonance are the highest.

Since the emission is significantly enhanced when the Mie resonances are excited, the power radiated in free space *P*_rad_ not only originates directly from the sound source, but also from the maze-like antenna itself. To verify this point, we show in Fig. 2(d–f) for each mode the polar plot of simulated and experimentally measured far-field sound radiation pattern normalized by the monopole source without the antenna. Three remarks should be noted: (1) The radiation pattern of R1 in Fig. 2(d) shows a suppression between 0° ± 90° as the power directed by the antenna is less than that of the monopole source. (2) One can see in Fig. 2(e,f) that the radiation patterns of R2 and R3 are very different and more directive than the monopolar distribution. For the resonance R2, the antenna excites the bidirectional radiation pattern and the sound intensity is larger than that of the monopole source only between $60^\circ  \sim 110^\circ$ and $250^\circ  \sim 300^\circ$. The max value of the intensity reaches 1.4 times of the value for the monopole source. (3) For the resonance R3, we observe the unidirectional radiation characteristics in the direction of $0^\circ$ as the sound intensity is 7.81 times larger than that of the monopole source. The half-power beam-width (HPBW) of the main lobe is $68^\circ$, which is defined as the angle between the two directions in which the radiation intensity is one half-value of the beam. The difference between the intensity of the main lobe and that of the side lobes is about 5.3 times. By comparing the different plots shown in Fig. 2(d–f), we conclude that the R2 and R3 resonances can assist the monopole source to yield more power radiated into free space and hence a better directivity. To quantify the directional characteristics, we also calculate the directivity *D* of the antenna at each resonance mode, which can be written as *D* = 2π*P*(*θ*)/*P*_rad_ in two-dimensional cases. *P*(*θ*) is the power per unit solid angle emitted in the given direction. The maximal directivities *D*_max_ = max(*D*) for resonance R1, R2 and R3 are 1.49 dB, 2.92 dB and 5.05 dB, respectively.

### Construct array antenna to optimize performance

**Figure 3:**
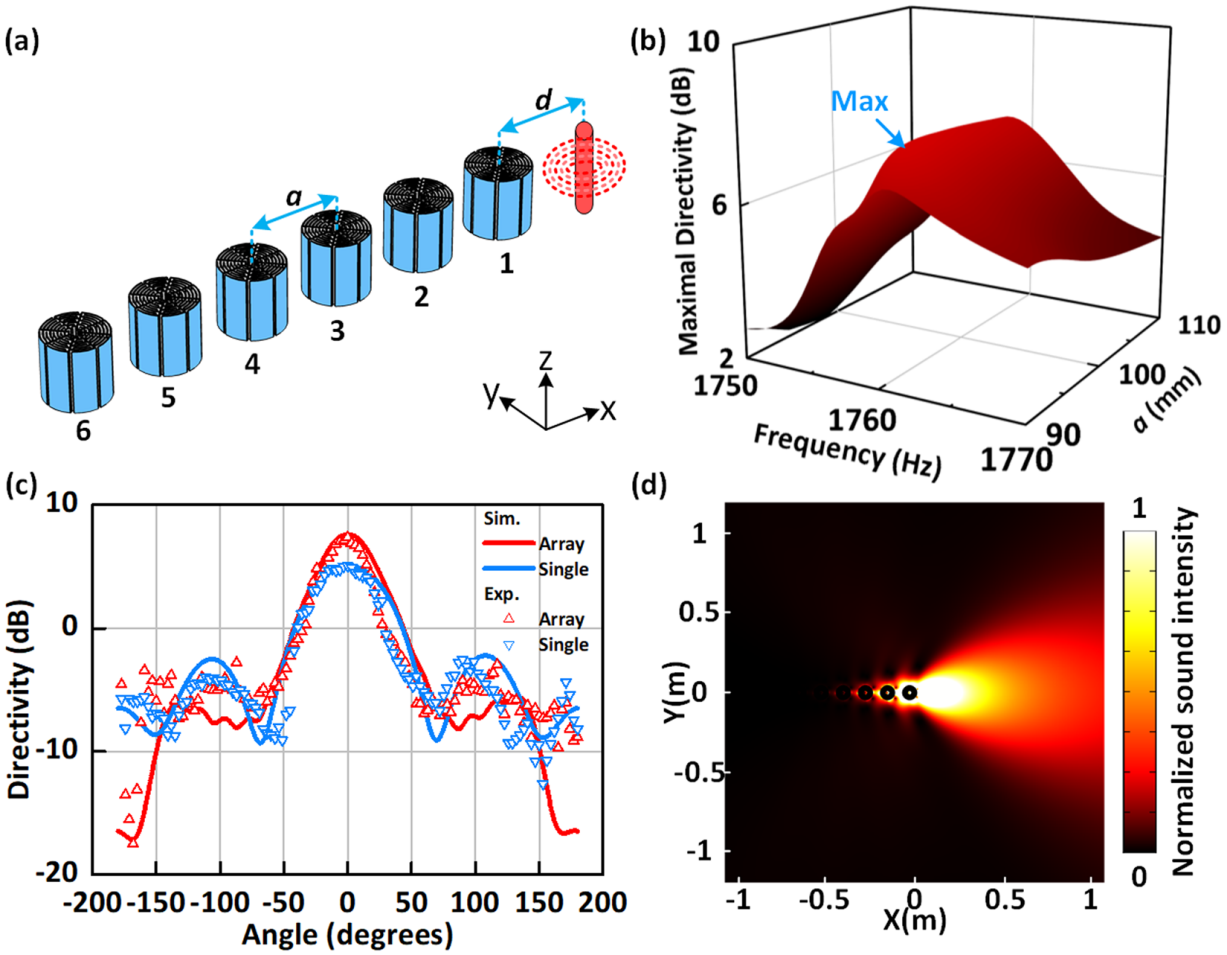
Array antenna consists of the several Mie resonators. (**a**) Schematics of the array antenna consisting of 6 Mie resonators. (**b**) Maximal directivity of the array antenna as a function of the frequency *f* and interval distance *a*. (**c**) Simulated and measured directivity of the array antenna (red line and scatters) and the single resonator antenna (blue line and scatters) in linear plot. (**d**) Field distributions of sound intensity in the *xy*-plane at resonance R3 for the array antenna.

By constructing array antenna with several Mie resonators [see Fig. 3(a)], we demonstrate the improved enhancement of intensity in the main lobe and concentration of the radiation energy in the wanted direction. In addition to the coupling between the Mie resonator and the sound source, the coupling between adjacent resonators also affects the radiation pattern. For illustration, we plot the maximal directivity *D*_max_ as a function of frequency *f* and interval distance *a* around resonance R3 in Fig. 3(b). The antenna exhibits the strongest directivity *D*_max_ = 7.62 dB when the distance is set as *a* = 96 mm and the corresponding resonant frequency is 1759 Hz. The increase in the number of coupling unit causes a slight offset in the resonant frequency. For the antenna consisting of 6 Mie resonators, the normalized sound intensity in the direction of $0^\circ$ can reach the value of 8.93 compared to the value of 7.81 for a single resonator. We have also investigated the effect of resonator number on the antenna performance (see Supplementary Note. 1), and the increase in the number of units will improve the directivity of the antenna and the improvement is ignorable when the number is more than 6. The linear plot of the simulation and experiment results of the directivity *D* in Fig. 3(c) are consistent and clearly confirms the optimization of the directivity: the amplitude of the main lobe increases while that of the side lobes is suppressed. The difference between the intensity of the main lobe and that of side lobes reaches about 22.3 times. Besides, the half-power beam-width is reduced from $68^\circ$ to $48^\circ$, indicating that the radiated power is much more concentrated. The distribution of the intensity in the *xy*-plane at resonance R3 for the array antenna [see Fig. 3(d)] gives an intuitive display of the unidirectional radiation performance.

### Tunability of the radiation direction

**Figure 4:**
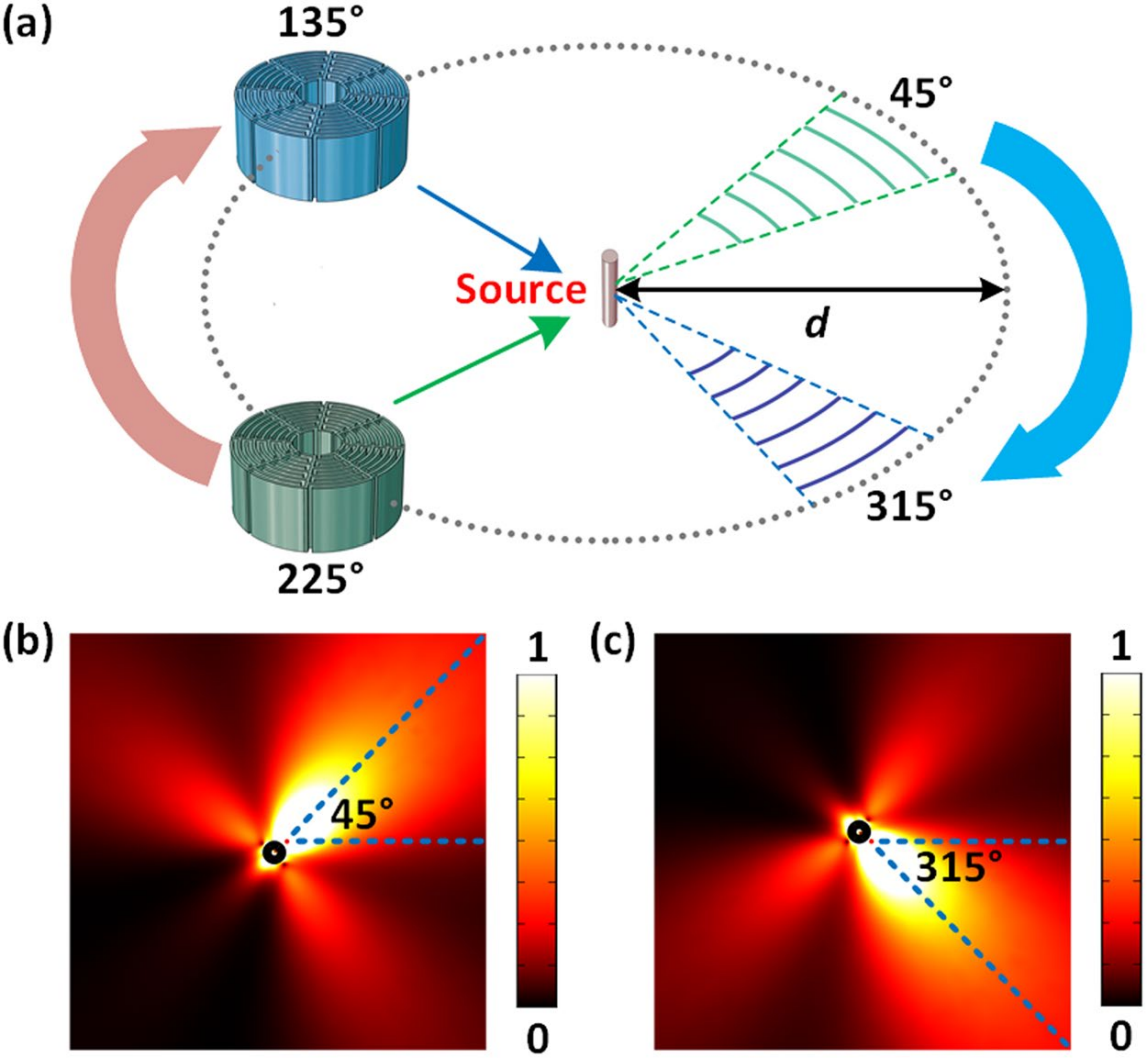
Tunability of the radiation direction (**a**) Schematics of the reconfigurable antenna. The Mie-resonant unit is physically rotated in a subwavelength scale to sweep the directional beam through the area of interest. The antenna (marked in green) is placed at the angle of 225° originally and the sound energy is radiated to the direction of 45°. After rotating the antenna (marked in blue) to the angle of 135°, the radiation direction shifts to 315° correspondingly. The corresponding distributions of the sound intensity for the two cases are shown in (**b**) and (**c**), respectively.

Traditional methods to adjust the directivity of sound radiation require devices with physical dimensions comparable to the wavelength, and it is hard to configure the radiation direction due to the complexity of the systems. The antenna presented in this paper has its advantage of subwavelength scale and configuration flexibility. As schematically shown in Fig. 4(a), to achieve structural tunability, computer-controlled motor is attached to the pedestals of the Mie resonator to provide the desired rotation. Thus, the antenna rotates around the point source in a circular orbit of a radius *d* and the radiation direction changes accordingly. The radius *d* = *λ*/4.89 at the resonance frequency of 1755Hz so that the rotation is in a sub-wavelength scale. Two different configurations are illustrated [see supplementary movie for the animation showing the radiation pattern modification of the Mie-resonator antenna as the radiation angle of the sound beam is swept from $0^\circ$ to $360^\circ$]: when the antenna is placed at the angle of 225° (marked in green), the strongest radiation direction is shifted to 45°. The radiation direction can be switched to 315° if we rotate the antenna to the angle of 135° (marked in blue). Figure 4(b,c) exhibit the distributions of the sound intensity in the *xy* plane corresponding to the two different cases. Therefore, we can sweep the beam through the area of interest and the selectable beam direction is easy to be controlled. Thus, the simplicity and flexibility of the antenna provide more possibilities for its potential applications.

### Wide applicability of the acoustic antenna

**Figure 5:**
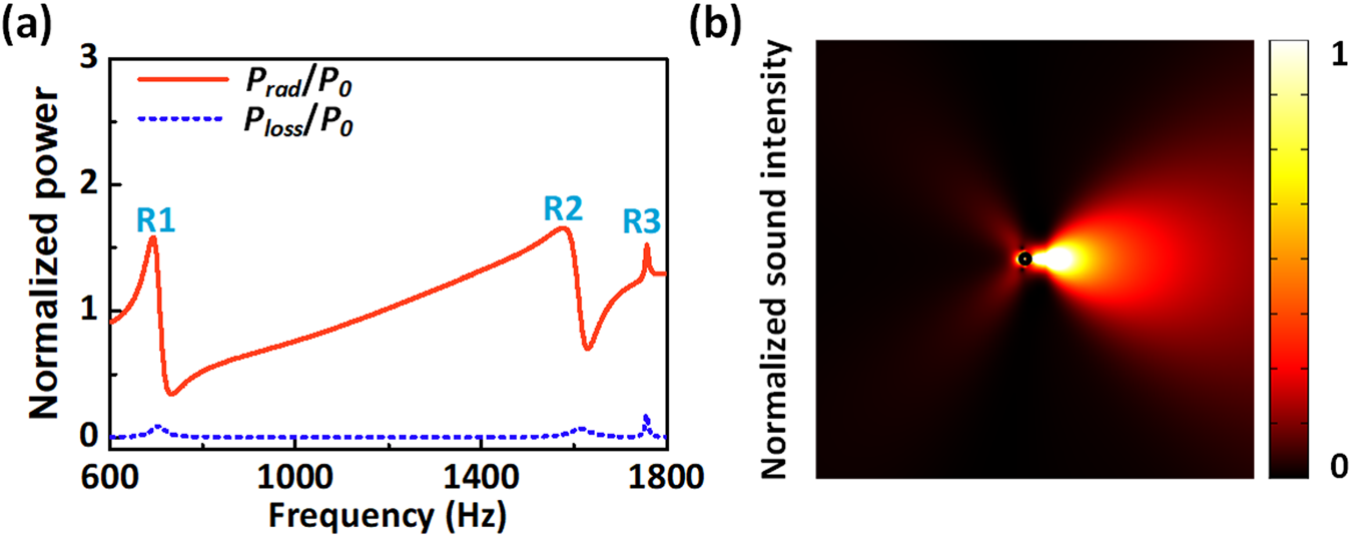
Wide applicability of the acoustic antenna. (**a**) Normalized radiated power and dissipated power for the Mie-resonance-based antenna coupled to a dipole source. (**b**) Simulated field distributions of sound intensity in the *xy*-plane at resonance R3 for the antenna coupled to a dipole source.

Finally, to demonstrate that the proposed antenna can produce enhanced directional sound emission under different types of sound source, we depict the simulated radiation power spectrum in the case of coupling to a classics dipolar sound source in Fig. 5(a). The power spectrum also exhibits three peaks corresponding to the monopole mode, dipole mode and quadrupole mode of the Mie resonator, and the radiation efficiency is enhanced by about 50%. For the quadrupole mode R3, the antenna achieves unidirectional radiation as shown in Fig. 5(b). The sound intensity of the antenna in the direction of 0° reaches 3.5 times of the value for the dipole source and the half-power beam-width is 68°. Besides, the antenna shows an obvious suppression in the opposite direction. The effective modulation on the dipole source further suggests the wide applicability of the antenna in the modulation of classic sound sources.

## Discussion

In conclusion, we have constructed a sub-wavelength acoustic antenna which can emit enhanced directional sound beams. The design is beneficial from the intensive coupling between classic acoustic source and Mie resonances with a maze-like resonator. Such an acoustic antenna demonstrates a number of key advantages, including much enhanced emissivity, high directivity, easy tunability and wide applicability. Moreover, the array antenna composed of several resonators can further improve the unidirectional performance. Although the single resonator has its every dimension at an order smaller than the working wavelength, however, the radiated power is improved up to 2.33 times of the direct radiation of a monopole source and the sound intensity received in the main lobe direction can be enhanced by 7.81 times. The enhancement of the sound intensity can reach the value of 8.93 in the case of the antenna array consists of 6 resonators, with a length *L* ≈ 2.8λ. This acoustic antenna may provide new possibilities for the design of simple sound devices with high directivity.

## Methods

### Experimental setup

**Figure 6:**
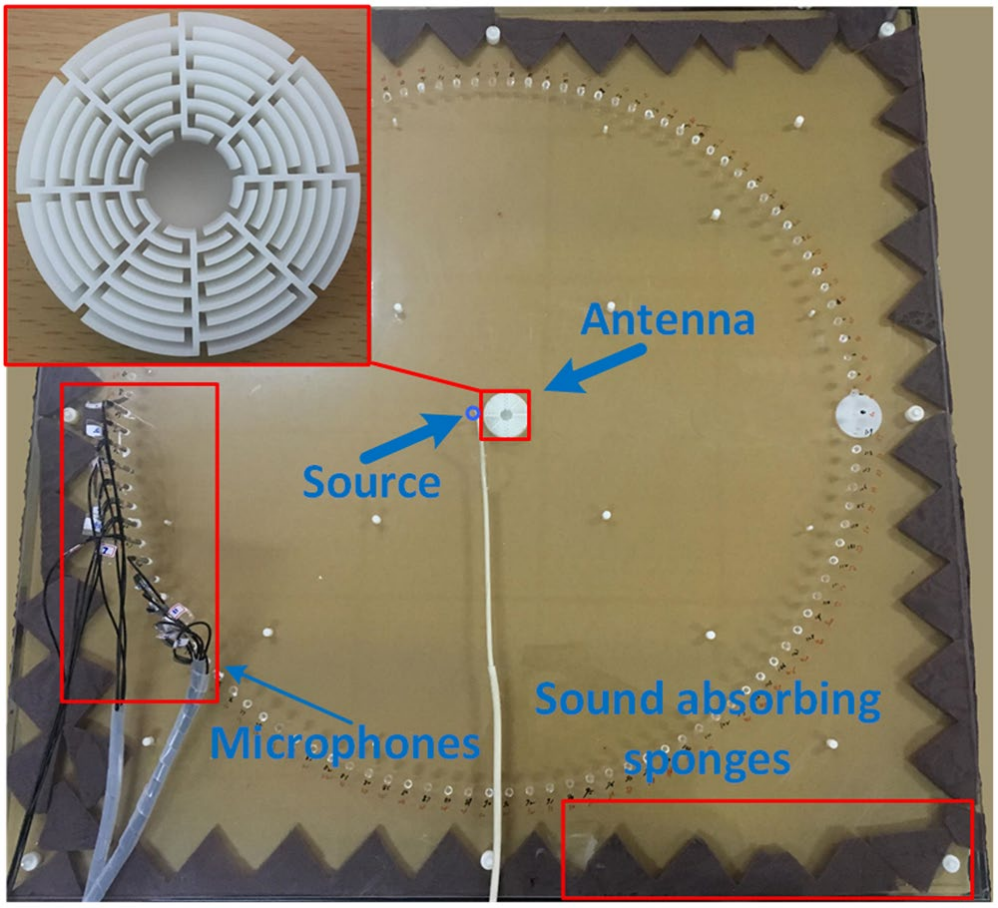
Schematic of the experimental set-up. Microphones are attached at the upper surface of the waveguide to measure the pressure fields. Inset: zoomed-in photograph of the fabricated Mie resonator sample.

The designed acoustic antenna is made of photosensitive resin and is manufactured via three-dimensional printing technology. Figure 6 shows the experimental set-up of the pressure field measurement. Input signals launched by the loudspeaker propagate through the hose and radiate sound waves at the center of the waveguide. The radius of the hose is *R*_*h*_ = 7.1 mm and is much smaller than the wavelength. The maze-like antenna is placed beside the source and then the signals are recorded by 15 1/4 inch condensed microphones (Brüel & Kjær type-4939). Sound absorbing sponges are placed around the waveguide to eliminate the effects of reflected waves.

### Numerical simulations

To characterize the radiation efficiency of the sub-wavelength antenna, we perform full wave simulations with the commercial COMSOL Multiphysics 5.3a software based on the finite element method. The density and sound speed of artificial structure are set as *ρ* = 1050 *kg/m*^3^ and *c* = 2200 *m*/*s*, respectively. The background material is air with *ρ*_*air*_ = 1.21 *kg*/*m*^3^ and *c*_*air*_ = 343 *m*/*s*. Perfectly matched layers (PMLs) are imposed on the outer boundaries of simulated domain to eliminate the interference from reflected waves.

### Data availability

The datasets generated during and/or analyzed during the current study are available from the corresponding author on reasonable request.

## Electronic supplementary material

Supplementary material

Supplementary Movie
