# Supplementary material for: Tunable directional subwavelength acoustic antenna based on Mie resonance
Source: Sci Rep. 2018 Jul 3;8:10049. doi: 10.1038/s41598-018-27970-5 (PMC6030154; doi:10.1038/s41598-018-27970-5)
Supplement: Supplementary file 1 — Supplementary material [file 41598_2018_27970_MOESM1_ESM.pdf]

Supplementary Material for the paper

“Tunable directional subwavelength acoustic antenna based on Mie resonance”

Jin Zhang[1], Ying Cheng[1,2], Xiaojun Liu[1,2]

[1]Key Laboratory of Modern Acoustics, Department of Physics and Collaborative Innovation Center of Advanced Microstructures, Nanjing University, Nanjing 210093, China [2]State Key Laboratory of Acoustics, Institute of Acoustics, Chinese Academy of Sciences, Beijing 100190, China.

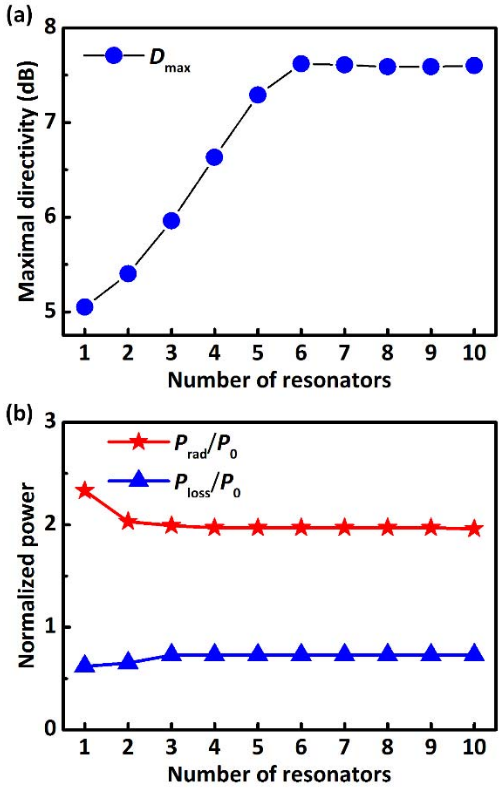

Supplementary figure 1: Influence of the resonator number on the antenna performance. (a) Simulated maximal directivity of the antenna. (b) Simulated normalized radiated power (marked in red pentagram) and dissipated power (marked in blue triangle) of the antenna system.

Note 1. Influence of the resonator number

As shown in supplementary Fig. 1(a), the increase in the number of the resonators will improve the directivity of the antenna. As the number of resonators increases from 1 to 6, the maximum directivity D_{max} changes from 5.05 dB to 7.62 dB. When the number of resonators is more than 6, the antenna directivity tends to be stable. Therefore, the optimal number of the resonators can be chosen as 6, which promise the best directivity and small antenna size at the same time. In addition, we have also investigated the influence of the resonator number on the radiated and dissipated power, as shown in supplementary Fig. 1(b). As the resonator number increases, the variation of radiated power and loss power is small, compared to the significant increase in directivity. Thus, the array antenna exhibit improved overall performance than a single resonator antenna.
